# Factors associated with implemented teacher-led movement and physical activity in early childhood education and care

**DOI:** 10.3389/fpsyg.2023.1221566

**Published:** 2023-07-27

**Authors:** Ann-Christin Sollerhed

**Affiliations:** Faculty of Teacher Education, Kristianstad University, Kristianstad, Sweden

**Keywords:** early childhood, motor skills, motor development, physical activity, teacher led activity, free play

## Abstract

Movement and physical activity (MoPA) is critical for children’s development and health. This study aimed to explore early childhood education and care (ECEC) educators’ reported frequency of implemented gross motor and physical activities (MoPA) among children in ECEC, as well as the educators’ reported personal physical activity (PA) levels in leisure time. A cross-sectional survey was performed in 68 preschools in southern Sweden. Data were obtained from questionnaires completed by 359 ECEC educators. The participation rate was 61%. About two thirds offered MoPA once a week or more seldom, while one quarter offered MoPA at least every other day. Educators who reported personal PA three times or more per week, offered MoPA for the children at least every other day to a higher extent (37%) compared to colleagues who reported personal PA once or twice a week (26%) or colleagues who reported that they were never or seldom active (18%) (*p* = 0.034). The results from multiple logistic regression analysis showed that reported implemented MoPA among children in ECEC was significantly associated with the educators’ perceptions that free play improved children’s gross motor skills (OR 2.7), the educators’ perceptions of needed curricular guidelines for MoPA (OR 2.1), the educators’ own leisure PA level (OR 2.0) and the educators’ perceptions that adequate gross motor skills were not learned at home (OR 0.4). Teacher-led MoPA occurs sparingly during the preschool day and the teachers believe that the children get sufficient MoPA in free play. The children are expected to develop their motor skills to a sufficient extent during the short moments of offered outdoor play. Teachers who are physically active in their leisure-time seem to offer gross motor training for the children to a higher extent than less active or inactive colleagues.

## 1. Introduction

The 21st century has seen steady growth in early childhood education and care (ECEC) to support parents with young children’s education and care (Cohen and Korintus, 2017), and ECEC is the first step into the formal education system (Broström et al., 2018). In 2021, 86% of children of preschool age (1–5 years) in Sweden participated in ECEC. On average, children spent 31 h a week in ECEC (Skolverket, 2021).

As most children spend a large proportion of their waking hours in ECEC, the educators are important for promoting, teaching, and modeling children’s physical activity (PA) (Mavilidi et al., 2021), which in turn is important for children’s motor development and to ensure they have the daily recommended PA. Early childhood development lays the foundation for a lifetime’s mental and physical health, education, labor market productivity, and well-being (Shonkoff, 2014; Richter et al., 2020). PA offers significant physical health benefits in children, and prevents chronic diseases such as cardiovascular disease, diabetes, or osteoporosis later in life (Carson et al., 2017). Moreover, there seems to be a significant association between PA and cognitive and academic performance in children. PA improve physical self-perceptions and enhance self-esteem, which seem to be mechanisms that affect cognitive and mental health in children (Lubans et al., 2016). Activities with physical exertion (high intensity) seem to have the strongest effects on cognition, specifically on executive functions (Vorkapic et al., 2021).

Participation in PA among children under the age of five has declined globally in recent years, while childhood obesity is, worryingly, increasing (World Health Organization, 2019). The WHO recommends at least 180 min of PA per day at any intensity level, including at least 60 min of moderate-to-vigorous physical activity (MVPA), and reduced time sitting for children under the age of 5 years (World Health Organization, 2019). ECEC is regulated by governing documents for example in law and curriculum. The responsibility to organize pedagogical activities to promote the principles stated in the curriculum lies with the educators, yet specific pedagogical methods are not identified in the curriculum (Einarsdottir et al., 2015). MoPA was found to have a low priority, to varying degrees, in the ECEC policies enacted by Nordic countries, and the guidance provided to educators and stakeholders therein was inexplicit (Sollerhed et al., 2021), which may mean that educators do not implement MoPA for young children in ECEC.

Fundamental Movement Skills (FMS), defined as basic learned movement patterns, which include gross motor skills such as stability, locomotor skills, and object control (Barnett Lai et al., 2016), are vital for motor development in children (Eddy et al., 2019). The FMS can be regarded as building blocks for movement and are essential for learning more complex skills (Clark and Metcalf, 2002), which in turn may lead to higher PA levels (Hardy et al., 2010a; Lubans et al., 2010), especially in MVPA (Williams et al., 2008).

Various types of teachers’ strategies and qualifications to handle MoPA have been identified in studies as well as MoPA-related outcomes among children (Mak et al., 2021). Teacher training in MoPA practice is essential to deliver adequate MoPA for children in ECEC (Vanderloo et al., 2014; Mavilidi et al., 2021). The ECEC educators meet children who are in a period of rapid brain development, growth, and motor development (Shonkoff, 2014), a period when also healthy behaviors are established (Ward et al., 2010). ECEC educators are agents who create conditions for children’s learning in preschool practice (Dalli et al., 2012). This includes the teaching of adequate MoPA, which is essential for MVPA levels (Barnett Lai et al., 2016). Adequate teacher led programs have been shown to have the potential to improve motor skills in young children (Adams et al., 2009; Hardy et al., 2010b; Van Cauwenberghe et al., 2013). An adequate education in teaching MoPA is important. Teacher training could positively influence perceptions and attitudes to increase the professional educator’s perceived competence when implementing PE in ECEC (Soini et al., 2021).

Parents often think that children are highly active in preschool, and thus offer few opportunities for MoPA at home (Pate et al., 2008), but children were observed to be physically inactive during most of their time in ECEC, and physically active in MVPA only 2–3% of the time (Pate et al., 2008; Soini et al., 2014). Sedentary time has been shown to be high in ECEC (Reilly, 2010). Another study showed that mean MVPA in preschool children was about 16 min per day. It takes at least twice that amount to achieve the positive effects of bone mineralization (Raustorp et al., 2012).

The social and physical environment in ECEC has an important influence on children’s MoPA (Brown et al., 2009a). The educators are role models, and they need adequate competence in MoPA to provide opportunities for children to learn motor skills. Children’s motor skills have been shown to lead to better immediate and long-term health and education outcomes (Shonkoff, 2014). The educators’ behavior and attitudes are the least studied correlates when it comes to correlates of children’s MoPA and/or sedentary behavior in ECEC (Tonge et al., 2016).

Therefore, the aim of this study was to explore ECEC educators’ reported frequency of implemented gross motor and physical activities (MoPA) among children in ECEC during the preschool day, their opinions about learning situations for motor skills in ECEC, as well as the educators’ reported personal PA levels in leisure time. Additional aims were to explore the educators’ perceived competence level to teach MoPA and how they perceived the occurrence of guidelines for MoPA in the policy documents for ECEC.

## 2. Methods and materials

### 2.1. Participants

The study took place in 68 preschools in southern Sweden. The sample represented preschools from the countryside and mid-size municipalities. The preschool units consisted of both mixed and single age groups. On average, teacher-child ratios were 1:5 with ranges of 1:3 to 1:8. Three hundred fifty-nine in-service ECEC educators completed the self-report questionnaire (response rate 61%). The participants (aged 20–65 years old) worked as educators in ECEC and were either preschool teachers or day-care attendants. About half of all ECEC employees in Sweden are preschool teachers that have 3.5 years of university training and the other half are day-care attendants who have upper-secondary qualifications (Sandberg and Ottosson, 2010).

### 2.2. The survey

A questionnaire was distributed to individual ECEC educators to obtain statistically useful information about their experiences and perceptions about MoPA. A literature search did not show any previous studies related to MoPA in ECEC in Sweden and therefore the questions of the current questionnaire were developed by the research group specifically for the aim of this study. The questions in the questionnaire were developed from the perspective of content in the curriculum for ECEC, completed with questions about PA behavior that have been used in other studies (Sollerhed, 2006). The questionnaire was piloted with 47 preservice teachers in ECTE outside the study before it was used in the actual survey. The questionnaire consisted of either multiple-choice questions or Likert scale questions. The questions were distributed in three parts: (1) Participants’ background information; (2) Participants’ experiences and perceptions of their own PA behavior; (3) Participants’ experiences and perceptions of MoPA in ECEC. Participants were asked to evaluate their experiences and perceptions of different activities and aspects in ECEC on a scale from 1 = completely disagree to 4 = completely agree. The teachers’ perception of children’s MoPA at home was also investigated in a similar Likert scale question. The data collection was performed at each preschool and the participants filled in the survey on paper individually in connection to their working day.

### 2.3. Statistical analysis

SPSS Statistics version 28 software was used to carry out both descriptive and analytical statistics. Data were first analyzed using descriptive statistics with frequencies and percentages. Secondly, Chi-square tests were used to investigate any associations between the frequency of implemented gross motor teaching in the preschools and the independent variables of educators’ personal MVPA, perceived education level in MoPA, perceived competence to teach MoPA, perceptions of children’s learned motor skills at home, at preschool and in free play, perceptions of the actual curriculum, wish for guidelines for MoPA, and enjoyment of teaching MoPA.

Finally, multiple logistic regression analysis was conducted to examine any association between the described independent variables and the likelihood of reported implemented gross motor teaching among children in the preschool. The rationale for using a logistic regression analysis is that it allows us to see the effects of variables after adjusting for other variables. This helps us to see and verify if the associations seen in the bivariate analysis are due to the influence of other variables. The method “enter” was used in the logistic regression analysis, which is a procedure for variable selection in which all independent variables are entered in a single step. The assumption of absence of multicollinearity was examined among the variables by calculating variance inflation factors (Menard, 2010). The responses in dependent and independent variables were collapsed and dichotomized before the multiple logistic regression analyses. The coding and dichotomizing of response options are shown in Table 1. The significance level was set at *p* < 0.05.

**Table 1:** Dichotomized variables included in the logistic regression with reported frequency of implemented gross motor skills training as the dependent variable.

Item	Response options	Dichotomization
Reported personal PA “How often do you exercise in your free time for at least half an hour so that you become short of breath and sweaty?”	7 categories Never (1) → > 4 times/week (7)	PA ≥3 times/week (6–7) PA 2 times or less/week (1–5)
Adequate MoPA in free play “Children learn adequate gross motor skills in free play.”	4 categories Totally agree (1) → Totally disagree (4)	Totally agree (1) Disagree to some extent or totally disagree (2–4)
Adequate MoPA at home “Children learn adequate gross motor skills at home.”	4 categories Totally agree (1) → Totally disagree (4)	Totally agree (1) Disagree to some extent or totally disagree (2–4)
Adequate MoPA learning in ECEC “Children learn adequate motor skills through organized teaching in preschool.”	4 categories Totally agree (1) → Totally disagree (4)	Totally agree (1) Disagree to some extent or totally disagree (2–4)
Curriculum supportive for MoPA “The curriculum provides sufficient support for gross motor training in preschool.”	4 categories Totally agree (1) → Totally disagree (4)	Totally agree (1) Disagree to some extent or totally disagree (2–4)
Adequate competence for MoPA teaching “I have adequate competence to lead and teach gross motor skills training.”	4 categories Totally agree (1) → Totally disagree (4)	Totally agree (1) Disagree to some extent or totally disagree (2–4)
Adequate education for MoPA teaching “I have an adequate education for work with gross motor skills teaching.”	4 categories Totally agree (1) → Totally disagree (4)	Totally agree (1) Disagree to some extent or totally disagree (2–4)
Guidelines for MoPA in ECEC “I am positive towards guidelines for adequate gross motor training for children in preschool.”	4 categories Totally agree (1) → Totally disagree (4)	Totally agree (1) Disagree to some extent or totally disagree (2–4)
Enjoy teaching MoPA “I enjoy and find pleasure in teaching children gross motor skills.”	4 categories Totally agree (1) → Totally disagree (4)	Totally agree (1) Disagree to some extent or totally disagree (2–4)

### 2.4. Ethical consideration

The study was conducted following the ethical principles for research involving human subjects, and all procedures were in accordance with the Declaration of Helsinki and the Swedish law on research ethics (SFS:2003:460). Ethical review application was approved by the Regional Ethical Review Committee in Lund (Dnr:2017/555). The participants were informed about the study, their voluntary status, and confidentiality, and informed written consent was obtained from all participants.

## 3. Results

As shown in Table 2, about one quarter of the educators reported that they offered gross motor skills training for the children every other day or every day, while about two thirds of the educators reported that they offered gross motor training once a week or more seldom. About a fifth of the educators reported that they offered MVPA every other day or every day, while four fifths reported MVPA once a week or more seldom. The reported personal level of MVPA differed between the educators. About 30 percent of the educators reported MVPA very seldom or never, while about 40 percent reported regular MVPA once or twice a week and about 30 percent reported MVPA three or more times a week (Table 2).

**Table 2:** Distribution of ECEC educators’ answers to questions and statements in the questionnaire.

Item	Options	Frequency *n* (%)
Planned gross motor training “How often does planned teacher led gross motor training occur in your preschool?”	Every day Every other day Once a week More seldom than once a week	57 (16.9) 37 (10.9) 185 (54.7) 59 (17.5)
Planned MVPA “How often does planned MVPA occur in your preschool?”	Every day Every other day Once a week More seldom than once a week	39 (11.7) 30 (9.0) 160 (48.2) 100 (30.1)
Spent time outdoor “How often do the children spend time outdoors?”	Every day Every other day Once a week More seldom than once a week	346 (98.9) 2 (0.6) 2 (0.6) -
Walking a little longer distance “How often does the group of children walk a longer distance?”	Every day Every other day Once a week More seldom than once a week	11 (3.2) 38 (11.1) 202 (59.2) 90 (26.5)
Categories of staff members “What is your highest education?”	Day-care attendant Preschool teacher Other	107 (30.2) 206 (58.2) 41 (11.6)
Reported personal PA “How often do you exercise in your free time for at least half an hour so that you become short of breath and sweaty?”	Never A few times a year A few times a month Regularly once a week Regularly twice a week Regularly three times a week Regularly four times or more a week	10 (2.8) 23 (6.4) 66 (18.6) 59 (16.5) 84 (23.5) 71 (19.9) 44 (12.3)
Adequate MoPA in free play “Children learn adequate gross motor skills in free play.”	Totally agree Partially agree Partially disagree Totally disagree	218 (62.3) 128 (36.6) 2 (0.6) 2 (0.6)
Adequate MoPA at home “Children learn adequate gross motor skills at home.”	Totally agree Partially agree Partially disagree Totally disagree	58 (17.7) 214 (65.2) 52 (15.9) 4 (1.2)
Adequate MoPA learning in ECEC “Children learn adequate motor skills through organized teaching in preschool.”	Totally agree Partially agree Partially disagree Totally disagree	214 (61.3) 127 (36.4) 7 (2.0) 1 (0.3)
Curriculum supportive for MoPA “The curriculum provides sufficient support for gross motor training in preschool.”	Totally agree Partially agree Partially disagree Totally disagree	99 (29.5) 176 (52.4) 51 (15.2) 10 (3.0)
Adequate competence for MoPA teaching “I have adequate competence to lead and teach gross motor skills training.”	Totally agree Partially agree Partially disagree Totally disagree	223 (64.1) 118 (33.9) 6 (1.7) 1 (0.3)
Adequate education for MoPA teaching “I have an adequate education for work with gross motor skills teaching.”	Totally agree Partially agree Partially disagree Totally disagree	90 (28.7) 135 (43.0) 72 (22.9) 17 (5.4)
Guidelines for MoPA in ECEC “I am positive towards guidelines for adequate gross motor training for children in preschool.”	Totally agree Partially agree Partially disagree Totally disagree	189 (54.8) 133 (38.6) 19 (5.5) 4 (1.2)

The results of the bivariate analyses between reported implemented gross motor training in preschool and different variables are shown in Table 3. The results showed significant associations between the educators’ reported personal MVPA in leisure time and the reported frequency of allocated gross motor training for the children in the preschool. The educators who reported MVPA three times or more per week, reported that they offered gross motor training for the children at least every other day to a higher extent (37%) compared to colleagues who reported personal MVPA once or twice a week (26%) or colleagues who reported they were never or seldom active (18%) (*p* = 0.034). There was also a significant association between the educators’ opinion that children learn sufficient gross motor skills in free play and the reported frequency of allocated gross motor training (*p* < 0.001) (Table 3).

**Table 3:** Relationship between ECEC educators’ reported frequency of gross motor training in ECEC and reported personal PA, and opinions about MoPA.

	Gross motor training every day	Gross motor training every other day	Gross motor training once a week or more seldom	*P*-value*
Reported personal PA (%)				
3 times or more/week	20.4	16.8	62.8	**0.034**
1–2 times/week	16.8	9.5	73.7	
Seldom or never	12.5	5.7	81.8	
Adequate MoPA in free play (%)				
Agree	22.3	11.0	66.7	**<0.001**
Disagree	5.5	11.2	80.3	
Adequate MoPA at home (%)				
Agree	14.0	7.0	78.9	0.597
Disagree	15.2	11.3	73.5	
Adequate MoPA learning in ECEC (%)				
Agree	18.0	12.2	69.8	0.241
Disagree	12.5	9.4	78.1	
Adequate competence for MoPA teaching (%)				
Agree	20.1	11.0	68.9	0.061
Disagree	10.4	9.6	80.0	
Adequate education for MoPA teaching (%)				
Agree	18.0	11.2	70.8	0.721
Disagree	15.0	9.8	75.2	
Guidelines for MoPA in ECEC (%)				
Agree	17.6	12.2	70.2	0.241
Disagree	14.2	8.8	77.0	
Enjoy teaching MoPA (%)				
Agree	18.9	12.4	68.7	0.068
Disagree	12.2	6.8	81.0	

^*^Chi-square test. *P*-value in bold when significant.

The results from the multiple logistic regression analysis showed that reported implemented gross motor training among children in the preschool was significantly associated with the educators’ perception that free play improved children’s gross motor skills adequately (OR 2.7; CI 1.3–5.5), the educators’ perceptions of needed guidelines for MoPA in the ECEC curriculum (OR 2.1; CI 1.1–4.1), the educators’ reported personal leisure PA level (OR 2.0; CI 1.9–3.8) and the educators’ perception that adequate gross motor skills were not learned at home (OR 0.4; CI 0.2–0.9) (Table 4).

**Table 4:** Variables associated with the educators’ reported gross motor teaching in a multiple logistic regression analysis (*n* = 348–359).

Variable	*B*	SE	χ^2^	P*	OR	95% CI
Reported personal PA	0.709	0.318	4.958	**0.026**	2.03	1.089–3.794
Adequate MoPA in free play	0.990	0.368	7.226	**0.007**	2.693	1.308–5.544
Adequate MoPA at home	−0.918	0.425	4.660	**0.031**	0.399	0.174–0.919
Adequate MoPA learning in ECEC	0.396	0.342	1.339	0.247	1.486	0.760–2.905
Curriculum supportive for MoPA	−0.228	0.359	0.403	0.525	0.796	0.394–1.608
Adequate competence for MoPA teaching	0.240	0.384	0.391	0.532	1.272	0.599–2.700
Adequate education for MoPA teaching	−0.017	0.350	0.002	0.962	0.983	0.496–1.951
Guidelines for MoPA in ECEC	0.730	0.346	4.444	**0.035**	2.075	1.053–4.091
Enjoy teaching MoPA	0.032	0.398	0.007	0.935	1.003	0.474–2.252

*P-value in bold when significant. *χ*^2^(8) = 273.24, *p* < 0.001, Hosmer–Lemeshow, *p* = 0.414, Nagelkerke *R*^2^ = 0.125. Variance Inflation Factors (VIFs) were calculated to detect the presence of multicollinearity between independent variables. All independent variables in the regression model have VIFs of 1.01–1.06.

## 4. Discussion

The findings of our study among 359 ECEC educators, revealed that about two thirds of them reported that they offered organized MoPA sessions once a week or more seldom, while less than one fifth of the educators offered MoPA daily for the children. The question is why few occasions are offered in general. The answer seems to be multifactorial and complex. Other studies have shown different explanations for barriers to incorporating MoPA in ECEC. One explanation was shown to be the desire for educators to favor work for academic schooling over MoPA training (Reilly, 2010). Another explanation could be the educators’ lack of competence to teach MoPA. It has been shown in other studies that it could be due to the educators’ personal attitudes and self-efficacy in MoPA-related issues (Parks et al., 2007; Copeland et al., 2012; Webster et al., 2015), as well as insufficient pedagogical content knowledge to teach MoPA, fear of injury and their own low fitness levels (Sollerhed, 2023). Educators’ insufficient understanding of the value and benefits of MoPA, which contribute to overall development, including cognitive and academic achievements (Lu and Montague, 2016) also affects the implemented MoPA.

The playground and the outdoor environment, including spending time outdoors, are often pointed out as crucial for children’s MoPA (Bower et al., 2008), especially if the educators are asked about prerequisites for MoPA. The effects of educators’ underlying attitudes about MoPA and actions are seldom pointed out, especially not by the educators themselves. Individual educators make daily decisions about when and how MoPA should be integrated or denied for children in ECEC. The policy documents regulate ECEC activities and should guide educators in their work. MoPA was shown to be of low priority in the ECEC policies, and the governmental guidance provided to educators and stakeholders therein is inexplicit (Sollerhed et al., 2021), which can increase the impact from the educator’s attitudes, perceived competence and habitus. The importance of educators’ behaviors toward children’s MoPA, including positive or negative prompts and modeling, has been shown to be vital (Brown et al., 2009b).

In our study, it was shown that the educators’ statements that children learned motor skills sufficiently through free play in ECEC was associated with the reported level of implementation of MoPA. The educators can act either as facilitators for or barriers to children’s motor development. This cannot be ignored from a public health perspective. Children spend a significant amount of their waking hours in ECEC, and educators are key players in shaping young children’s active behavior, which is important not only for children’s actual development but also for lifelong healthy behavior. According to the ECEC educators’ answers in the questionnaire, the likelihood for reported implemented MoPA at least every other day was almost three times higher when the educators perceived that the children learned motor skills in free play. It can thus be interpreted that the implemented MoPA was usually in the form of free play and not in the form of intentional teacher-led activity. Free play might offer efficient MoPA for the children, but it is not a guarantee. If the children have access to an enriching play area, they might choose to be physically active, and the free play might give the children the possibility to develop motor skills and endurance capacity. On the other hand, children can choose to do sedentary activities instead and exclude MoPA and learning motor skills in the free play period. In short, free play does not guarantee the effects of MoPA and therefore it is important to have adequately led MoPA sessions to ensure that all children get a chance to develop FMS. Besides general motor development, in terms of health, the importance of increased PA for influencing children’s (0–5 years) adiposity, bone density and cardiometabolic factors must be emphasized (Timmons et al., 2012).

Several studies have shown that children generally spend less than 50 percent of a free play period in ECEC participating in MoPA (Verstraete et al., 2006), especially when the restrictions are comprehensive to exclude any risks. Structured and intentional MoPA sessions have been shown to substantially increase the total amount of children’s PA in comparison with the amount in free play (Frank et al., 2018), as well as to enhance the FMS (Barnett Lai et al., 2016). The reason for the educators’ strong beliefs that children are highly active in MoPA in free play and their choice to often let the children have free play instead of various teacher-led MoPA is unclear, but is probably related to their perceived insufficient competence to lead and instruct MoPA (Sollerhed, 2023), but may also be associated with indolence. In children’s free play, the educators often take the position of an inactive supervisor (Tandon et al., 2018), which might be perceived as a more convenient task compared to the position as an instructor with the responsibility to teach the children different techniques in MoPA. The educators may use free play perfunctorily and take the easiest way with the least energy-demanding effort for themselves. Adequate educator-led programs have the potential to improve motor skills in young children (Adams et al., 2009; Hardy et al., 2010b; Van Cauwenberghe et al., 2013), which assumes that the educators are more actively participating in the activities and are not just supervising them. Also the intensity of PA among the children heightens during teacher led FMS practice, especially when involving locomotor skills (Cliff et al., 2009; Kain et al., 2018).

In free play, children are given access to certain areas in the environment with or without restrictions. The educators are the gatekeepers to available play areas and maintainers of restrictions. There is a strong belief that children should be kept as safe as possible, and the increasing focus on safety affects restrictions on children’s freedom and possibility to develop motor skills in the play area. Outdoor play may offer great opportunities for children to use natural elements and may involve challenges, heights, and speed, including exploring the environment and taking risks.

As risky play is associated with injury it is highly limited with subsequent limited MoPA training (Brussoni et al., 2012, 2015; Bento and Dias, 2017). Risks of injury must be considered in ECEC, but the children’s living conditions must not be de-risked to the extent that they do not learn to develop their own risk assessment, which is a normal part of childhood and development (Wyver et al., 2010; Tremblay et al., 2015). In ECEC, children are under adult supervision most of the time and the adults frequently decide what children are allowed to do and where they are allowed to play (Kyttä, 2004). Thus, preschoolers are under the influence of the preferences and fears of the educators. Adult-imposed restrictions on risk-taking in free play and MoPA restrictions are often justified for safety reasons (Brussoni et al., 2015), with children being banned from a variety of experiences of MoPA and from accessing forbidden areas (Thomson, 2014). The reason behind heightened sensitivity to risk in children’s play is a fundamental professional dilemma experienced by practitioners on a day-to-day basis. There is a dilemma in dealing with children’s safety; on the one hand, it is important that the children do not suffer acute injuries, but on the other hand, it is important not to prevent the children’s participation and exploration in MoPA, which can affect their motor and physical development negatively in the longer term. Thus, daily practice is fraught with conflicting priorities between safety and development imperatives. The educators should be encouraged to allow children to experience risk in their play by evaluating the developmental benefits of risk-taking and by having increased awareness of the value of risk-taking (Sandseter, 2014). While the fear of injuries prevents MoPA in general, perceived lack of sufficient pedagogical content knowledge in MoPA prevents intentional MoPA sessions where FMS is trained (Sollerhed, 2022).

In our study, a high percentage of the educators were convinced that the children learned adequate motor skills in free play in ECEC, while far fewer were convinced that children learned adequate motor skills at home. As children engage in free play both in the preschool context and at home this seems to be a paradox. The study cannot explain this discrepancy.

An association between the educator’s reported personal PA level and the reported frequency of implemented MoPA for the children in ECEC was shown. The educators’ own interest in PA seems to be an important determinant of the possibility for children’s MoPA in ECEC. About one third of the participating educators in our study reported that they were physically active in their leisure time. The likelihood that intentional MoPA would be implemented for the children in ECEC was doubled if the educator reported personal PA in their own leisure time. Educators’ beliefs and experiences of their own PA could thus be seen as a possible explanation for integrated MoPA among children. It is positive that these physically active educators implement MoPA sessions for the children in ECEC, but on the other hand, it could be questioned if the occurrence of MoPA among children is due to educators’ personal attitudes. Many teachers value MoPA but lack confidence to teach it. Teachers may believe in the benefits of PA, but would rather teach other subjects (McBride et al., 2002; Morgan and Hansen, 2008).

Interestingly, educators’ own experiences and perceptions about PA seem to be determinants for promotion and teaching of MoPA in children in ECEC, despite existing curriculum. The educators’ actions are not always performed in accordance with the curriculum, which is the governmental guidance provided to educators. Human behavior and actions are not performed in accordance with rules, but are often based on mental states, such as beliefs, attitudes, desires, goals and intentions (Gibbs, 2001), and also in habitus and ingrained habits (Bourdieu, 2017). MoPA should be legitimized and implemented from the perspective of children’s development irrespective of educators’ own past experiences and habitus for PA. The educators’ wish for clearer guidelines for MoPA in the curriculum for ECEC was significantly associated with the reported implemented teacher led MoPA. The educators perceived that MoPA was insufficiently and vaguely mentioned in the curriculum, and they wished for more explicit guidance through the curriculum. MoPA was shown to be of low priority in the ECEC curricula (Sollerhed et al., 2021), which strengthened the educators’ reflections.

The strengths of this study are worth mentioning, but also the limits. The study had a relatively large sample size, and the response rate was acceptable. However, a skewness in the positive direction could be noted. The survey may have attracted those educators interested in MoPA while those who felt resistance toward MoPA did not participate to the same level. Despite a sense of being skewed in a positive direction, the results showed that MoPA seems to occur sparingly in ECEC and seems to depend on the teachers’ benevolence and self-interest. The study was a cross-sectional survey and was unable to establish causality. As a literature search did not show any previous studies related to MoPA in ECEC in Sweden the questions in the questionnaire were developed by the research group, which can be seen as a weakness. The questions were developed on basis of the perspective of the content in the curriculum for ECEC, which could be seen as a strength. The PA was self-reported and not objectively measured, which is another limitation. The questions about reported PA have been used in several studies before and have shown to have acceptable validation. The questionnaire was piloted outside the study before it was used in the actual survey.

## 5. Conclusion

The teacher led MoPA seem to occur sparingly during the preschool day and the educators believe that the children get sufficient PA and develop adequately the gross motor skills in free play. The children are expected to develop their gross motor skills adequately during the short moments of offered outdoor play. Educators who report that they are physically active in PA three times or more per week in their leisure-time seem to offer gross motor training for the children to a higher extent than less active or inactive colleagues. Thus, educators’ own experiences and perceptions about PA seem to be determinants for promotion and teaching MoPA in ECEC.

## Data availability statement

The raw data supporting the conclusions of this article will be made available by the author upon request.

## Ethics statement

The studies involving human participants were reviewed and approved by Regional Ethical Review Committee in Lund (Dnr:2017/555). The participants provided their written informed consent to participate in this study.

## Author contributions

A-CS contributed to the design of the study. A-CS contributed to the data collection and interpretation of the data, drafted and revised the manuscript, provided final approval, and agreed to be accountable for all aspects of the work to ensure integrity and accuracy.

## Funding

This research was sponsored by the Gyllenstiernska Krapperup Foundation (KR 2021-0045) and The Research Platform for Collaboration for Learning, Faculty of Teacher Education at Kristianstad University.

## Conflict of interest

The author declares that the research was conducted in the absence of any commercial or financial relationships that could be construed as a potential conflict of interest.

## Publisher’s note

All claims expressed in this article are solely those of the authors and do not necessarily represent those of their affiliated organizations, or those of the publisher, the editors and the reviewers. Any product that may be evaluated in this article, or claim that may be made by its manufacturer, is not guaranteed or endorsed by the publisher.
